# Associations between Nut Consumption and Health Vary between Omnivores, Vegetarians, and Vegans

**DOI:** 10.3390/nu9111219

**Published:** 2017-11-06

**Authors:** Rachel C. Brown, Andrew R. Gray, Siew Ling Tey, Alexandra Chisholm, Victoria Burley, Darren C. Greenwood, Janet Cade

**Affiliations:** 1 Department of Human Nutrition, University of Otago, P.O. Box 56, Dunedin 9014, New Zealand; siewling.tey@otago.ac.nz (S.L.T.); alex.chisholm@otago.ac.nz (A.C.); 2 Nutrition Society of New Zealand, Whanganui 4543, New Zealand; 3 Department of Preventive and Social Medicine, University of Otago, P.O. Box 56, Dunedin 9054, New Zealand; andrew.gray@otago.ac.nz; 4 Nutrition Epidemiology Group, School of Food Science and Nutrition, University of Leeds, Leeds LS2 9JT, UK; V.J.Burley@leeds.ac.uk (V.B.); J.E.Cade@leeds.ac.uk (J.C.); 5 Leeds Institute for Cardiovascular and Metabolic Medicine, University of Leeds, Leeds LS2 9JT, UK; D.C.Greenwood@leeds.ac.uk

**Keywords:** nuts, predictors, cardiovascular disease risk, chronic disease risk, diabetes, cancer, vegan, vegetarian, omnivore

## Abstract

Regular nut consumption is associated with reduced risk factors for chronic disease; however, most population-based studies lack consideration of effect modification by dietary pattern. The UK Women’s Cohort Study (UKWCS) provides an ideal opportunity to examine relationships between nut consumption and chronic disease risk factors in a large sample with diverse dietary patterns. Nut and nutrient intake from 34,831 women was estimated using a food frequency questionnaire among self-identified omnivores, vegetarians and vegans. In this cross-sectional analysis, higher nut consumption was associated with lower body weight (difference between highest and lowest consumption categories from adjusted model: 6.1 kg; 95% CI: 4.7, 7.6) body mass index (BMI, 2.4 units difference; 95% CI: 1.9, 2.9), and waist circumference (2.6 cm difference; 95% CI: 1.4, 3.8) (all *p* for linear trend < 0.001). Higher nut consumption was also associated with reduced prevalence of high cholesterol and high blood pressure; having a history of heart attack, diabetes and gallstones; and markers of diet quality (all adjusted *p* for linear trend ≤ 0.011). Higher nut consumption appeared overall to be associated with greater benefits amongst omnivores compared to vegetarians and vegans. Findings support existing literature around beneficial effects of nut consumption and suggest that benefits may be larger among omnivores. Nut promotion strategies may have the highest population impact by specifically targeting this group.

## 1. Introduction

Nuts are rich sources of cis-unsaturated fatty acids, fibre, vitamins, minerals, and a number of bioactive substances [1,2,3]. The regular consumption of nuts is associated with a reduction in all-cause mortality [4,5,6,7,8,9,10,11], and in particular cardiovascular disease (CVD) [4,5,6,7,8,9,10,11]. Some research has also shown that regular nut consumption is inversely associated with the incidence of cancer and diabetes, although these findings are less consistent [4,5,6,8,12,13,14,15,16].

While the health benefits of nut consumption are well documented, a number of studies have shown that nut intakes at the population level are far from ideal [17,18,19,20]. For example, data from the European Prospective Investigation into Cancer and Nutrition (EPIC) study showed that only 6.9% of the population consumed whole nuts on the day of the 24-h recall, with a mean population intake of 2.2 g/day [18]. This is similar to intakes reported in the United States of America (USA) [19] and New Zealand (NZ) [17], where the prevalence of whole nut intake was 6.0% and 6.9% respectively on a given day. Despite these low reported intakes, examining the predictors of nut intake is important to provide information for public health initiatives which aim to increase regular nut consumption as a means of reducing the risk of CVD and other chronic diseases. 

Only a small number of studies have previously examined predictors of nut consumption. In the USA [21] and NZ [17], nuts were most likely to be consumed by Caucasians with higher education and income levels. Likewise, in the EPIC cohort [18], education level was positively associated with nut intake. Age was also an important predictor of nut intake. In the USA, among those aged 19–51 years, the percentage of participants consuming nuts on a given day was 5.5%, compared to 8.4% among those aged over 51 years [21]. In NZ, the prevalence of whole nut consumption on a given day was highest among those aged 31–70 years [17]. These studies have provided useful information on which to base public health messages, but all are limited by the relatively low prevalence of nut intake, which limits the complexity of statistical models investigating plausible confounders, effect modifiers, and mediators.

The Seventh-Day Adventist study examined the associations between levels of nut consumption and coronary heart disease (CHD) for vegetarians and non-vegetarian, finding protective associations for both [22]. Identifying associations between nut intake and risk factors for chronic disease can also provide important information for strategies to reduce disease risk. Studies have consistently reported that regular nut consumption is associated with reductions in total and low-density lipoprotein (LDL) cholesterol [23]. The evidence for effects on blood pressure is less clear. Findings from NHANES 1999–2004 indicate that nut consumers had significantly lower systolic blood pressure and prevalence of hypertension [24]. However, in a cross-sectional analysis of a nationally representative NZ sample, there was no evidence for a difference in systolic or diastolic blood pressure between nut consumers and non-nut consumers [25]. Results from clinical trials also show mixed results, with the majority reporting no evidence of any effect on blood pressure [26,27,28,29]. 

A more consistent finding among different populations is that lower body weight, BMI and waist circumference are all seen among those who consume nuts in comparison to non-nut consumers. Epidemiological studies have found that nut consumers are leaner than non-nut consumers [30,31,32,33,34], which is in agreement with intervention studies which report that when nuts are added to the diet, weight gain is either negligible or less than predicted based on energy content [34,35,36,37,38,39].

Most of the aforementioned studies have included samples where dietary intakes do not vary widely, which can make generalising to the full range of intakes problematic, and have not been able to consider the potential effect modification of the associations by dietary patterns. The UK Women’s Cohort Study provides an ideal opportunity to examine a wide range of dietary patterns and the effects of nut intakes within these patterns. Due to the high percentage of vegetarians purposefully recruited into this cohort, nut consumption patterns are more diverse than seen in most other studies. Therefore, we aim to identify predictors of nut intake, and identify associations between nut consumption and chronic disease risk in a cohort of women with diverse dietary patterns.

## 2. Materials and Methods

### 2.1. Study Design and Participants

The UK Women’s Cohort Study (UKWCS) has been described in detail elsewhere [40]. In brief, the cohort was derived from responders to a mail survey of the World Cancer Research Fund, living in England, Wales, and Scotland. Responders willing to participate in a more detailed survey composed the population who were then contacted to participate in the UKWCS. Eligible participants included women aged 35 to 69 years (inclusive) who self-reported being a vegetarian or non-red-meat eaters. A group of non-vegetarians were selected by choosing for each vegetarian the next non-vegetarian from the stored direct mail database who was aged within 10 years of the vegetarian. From the 61,000 women contacted, 35,372 returned completed questionnaires, a response rate of 58%. Permission to carry out the baseline UKWCS was obtained by 174 local research ethics committees. 

### 2.2. Dietary Intake

Baseline dietary data were collected between 1995 and 1998 using a detailed self-administered 217-item postal food frequency questionnaire (FFQ) which was based on the European Prospective Investigation into Cancer and Nutrition (EPIC) study and asked about food intake over the last year. This FFQ was validated against a semi-weighed four-day record, and some biochemical measures [41], however, not for nut intake in particular.

### 2.3. Nut Intake

At baseline, total nut intake was derived from the FFQ which included items on almonds, cashews, peanuts, pecans, pistachios, and walnuts, but not including the items on nut butters or nut pates. Total intake of nuts was calculated by summing the intakes of nuts from the individual food items, which were determined by using the frequency categories to estimate weekly intake in terms of servings, which were then converted to servings per day. Amounts in g were then estimated using a serving size of 30 g [42]. Intakes in g per day were also grouped into consumption categories of ‘none’ (0 g/day), ‘less than one 30 g serving per week’ (less than 4.3 g/day), 2–6 servings per week’ (less than 30 g/day), ‘daily’ (30–59.9 g/day), and ‘more than daily’ (60 g/day or more). 

### 2.4. Lifestyle Questionnaire

Each participant completed a self-administered lifestyle questionnaire where information was collected on age, employment, education level, physical activity, ethnicity, menopausal status, age of menarche, parity, use of hormone replacement therapy (HRT) and oral contraceptive pill (OCP), smoking status, body weight, height, and waist circumference [43]. Participants self-classified as omnivores, vegetarians, or vegans. 

### 2.5. Statistical Analysis

For this cross-sectional analysis, participants were excluded if the data was biologically implausible i.e., they had extremely high (>6000 kcal/day or 25,080 kJ/day) (*n* = 80) or low (<500 kcal/day or 2090 kJ/day) (*n* = 6) energy intake, a BMI of less than 13 kg/m^2^ (*n* = 10) or implausibly high based on discordant height and weight measures (*n* = 2), a waist circumference of less than 50 cm (*n* = 25), or a height greater than 7 feet (213.36 cm) (*n* = 12). A total of 126 distinct participants were excluded from this analysis based on these criteria. We also only included women who provided information on nut consumption, thus a further 415 women were excluded. Thus, the final analysis was performed on 34,831 women.

Appropriate summary statistics were calculated for all measures of interest (participant characteristics, nut consumption, and measures of chronic disease risk), specifically means and standard deviations (SD) for approximately normally distributed continuous variables, medians and inter-quartile ranges (IQR) for non-normal continuous variables, and counts and percentages for categorical variables. Linear, binary logistic, and ordinal logistic regression models were used to identify associations with continuous, binary, and ordinal outcomes respectively. When ordinal predictor variables were investigated (nut consumption level), orthogonal polynomials were used to model linear and higher order trends. Linear trends are reported and while higher-order trends are noted, these are only discussed where they affect the interpretation of results (e.g., indicate non-monotonicity). Effect modification of associations between nut intakes and outcomes by dietary pattern (omnivore, vegetarian, and vegan) was investigated by adding an interaction between dietary pattern and each of the orthogonal polynomial terms for nut consumption levels. A Wald test was used to assess the overall significance of this interaction with pairwise comparisons performed only when this was significant. For continuous outcomes, where model residuals were skewed and/or heteroscedastic, log-transformations were investigated, and retained when this improved residual behaviour. For models using dietary pattern (omnivorous, vegetarian, and vegan), sensitivity analyses were performed using total meat servings per week. Analyses were conducted using Stata 13.1 (StataCorp LLC, College Station, TX, USA) and two-sided *p* < 0.05 was considered statistically significant.

## 3. Results

### 3.1. Participant Characteristics and Nut Consumption among the Cohort

The characteristics of all 34,831 women in the study are shown in Table 1, both overall and by nut consumption (none versus any). Mean age (SD) was 52.2 (9.3) years with a mean (SD) BMI of 24.5 (4.3) kg/m^2^. In general, the cohort had a low prevalence of smoking (11%), was well educated with over 27% with a degree, and over 50% were working in professional or managerial positions. The majority of the cohort reported being omnivores (*n* = 25,116, 72%), with over one-quarter being vegetarian (*n* = 9280, 27%), and 1% (*n* = 435) vegans. 

Overall 16% of respondents reported consuming no nuts, with the 84% of participants who consumed nuts comprising 52% of the sample estimated to consume nuts less than once per week, 28% 2–6 times per week, 3% daily, and 1% at least 2 times per day. 

Compared with non-nut consumers, nut consumers appear to be slightly younger (by four years), appreciably more physically active, leaner (by each of weight, BMI, and waist circumference), much more likely to be highly educated, with a higher proportion working in professional or managerial positions. Nut consumers also appear much more likely to be vegetarians and vegans, and consume around 10 % more energy and over two-thirds more alcohol. They also appeared to be more likely to be pre-menopausal, and use oral contraceptives, consistent with them being younger. No practically important differences were observed between these two groups in terms of height, smoking, age of menarche, or parity. The only substantial difference for ethnicity appeared to be amongst those classified as Asian who had around four times the odds of consuming nuts compared to those classified as White. It should be noted that the number of Asian participants was small. 

### 3.2. Predictors of Nut Consumption

Table 2 reports the unadjusted and adjusted odds ratios (OR) for frequency of consuming nuts (5 ordinal levels from none to more than daily) from ordinal logistic regression modes using selected demographic variables as predictors. Nut consumption levels were statistically significantly lower with greater age. Compared with those aged in their forties, those aged in their fifties, sixties and seventies were less likely to consume higher quantities of nuts. There was a statistically significant association between nut consumption and physical activity, with those who performed no vigorous physical activity less likely to consume higher amounts of nuts than those who performed some vigorous activity. Those working in professional positions were more likely to consume greater quantities of nuts compared to those in clerical or skilled positions. The percentage consuming nuts was statistically significantly greater for those with higher educational qualifications, where those with a degree had 2.26 times the odds (95% CI: 2.07, 2.46; *p* < 0.001) of consuming greater amounts of nuts compared to those with no education. Former smokers were more likely to consume higher quantities of nuts compared to both current smokers and those who have never smoked.

Both vegetarians (OR: 2.23; 95% CI: 2.12, 2.35; *p* < 0.001) and vegans (OR: 3.76; 95% CI: 3.05, 4.64; *p* < 0.001) were statistically significantly more likely to consume higher amounts of nuts compared to omnivores, In a model also adjusting for energy intake, this association remained and higher energy intakes were associated with greater likelihoods of consuming higher quantities of nuts (*p* < 0.001). 

### 3.3. Nut Consumption and Anthropometric Measures

In all unadjusted and adjusted models, for body weight, BMI and waist circumference there was evidence of a linear trend where higher nut consumption levels were associated with lower values (adjusted differences between the highest compared to lowest consumption categories, including total energy in the models: 6.1 kg (95% CI: 4.7, 7.6), 2.4 BMI units (95% CI: 1.9, 2.9), and 2.6 cm (95% CI: 1.4, 3.8), all *p* for linear trend < 0.001 (Table 3). 

### 3.4. History of Selected Chronic Diseases and Risk Factors for Chronic Disease by Nut Consumption

For two risk factors for chronic disease, namely, high blood pressure (for highest compared to lowest consumption category unadjusted OR: 0.54 (95% CI: 0.40, 0.76) and adjusted for variables other than BMI, which could be on the causal pathway as well as potentially a confounder, OR: 0.69 (95% CI: 0.48, 0.98) and high blood cholesterol (unadjusted OR: 0.57 (95% CI: 0.36, 0.90) and adjusted OR: 0.67 (95% CI: 0.40, 1.14)) there was evidence in all models for a linear trend where higher levels of nut consumption was associated with lower prevalence of these risk factors (all *p* for linear trend ≤0.010 for high blood pressure and ≤0.004 for high blood cholesterol) (Table 4).

For chronic disease, there was a statistically significant linear trend for all models for the prevalence of having a history of heart attack (unadjusted OR: 0.21 (95% CI: 0.05, 0.86), adjusted for variables other than BMI OR: 0.28 (95% CI: 0.07, 1.15)), diabetes (unadjusted OR: 0.40 (95% CI: 0.16, 0.99) and adjusted OR: 0.54 (95% CI: 0.20, 1.50)), and gallstones (unadjusted OR: 0.46 (95% CI: 0.26, 0.80) and adjusted OR: 0.46 (95% CI: 0.24, 0.92)) (all *p* for linear trend ≤0.011). 

For angina, although there was evidence of a linear trend for most models (all *p* for linear trend <0.016 without adjusting for BMI), when the model was further adjusted for BMI, the linear trend became a non-statistically significant tendency (*p* for linear trend = 0.092). A similar pattern was seen for stroke, where there was evidence of a linear trend in the unadjusted and age-adjusted model, which was no longer statistically significant after further adjustment (both *p* ≥ 0.115). 

For polyps and cancer, although there was evidence of a linear trend where higher levels of nut consumption was associated with lower prevalence of a history of these, in the unadjusted models (both *p* ≤ 0.003) and adjusting for age for polyps (*p* = 0.028), this was no longer statistically significant in the adjusted models (all *p* ≥ 0.119).

### 3.5. Nut Consumption and Nutrient Intake

As seen in Table 5, there was evidence of a linear trend for nutrients in all but one case for both unadjusted and adjusted models. Total energy, fibre, and the percentage of energy from total fat, monounsaturated fatty acids (MUFA) and polyunsaturated fatty acids (PUFA) were all higher amongst those with higher levels of nut consumption. The percentage of energy from saturated fat was lower amongst those with lower nut consumption in the adjusted model only. Likewise, the percentage of energy from protein, carbohydrate, and total sugar was lower amongst those with higher levels of nut consumption. 

For the majority of micronutrients (exceptions being vitamins A and C), there was statistically significant evidence of a linear trend in the models further adjusted for energy intake. However, both vitamin A and vitamin C demonstrated quadratic trends where, although intakes were higher with higher levels of nut consumption, the differences were less pronounced in successive nut intake categories. For most micronutrients, intakes were higher for greater levels of nut consumption, except for vitamin B12 which showed the opposite trend. 

### 3.6. Effect Modification by Dietary Pattern

**Figure 1:**
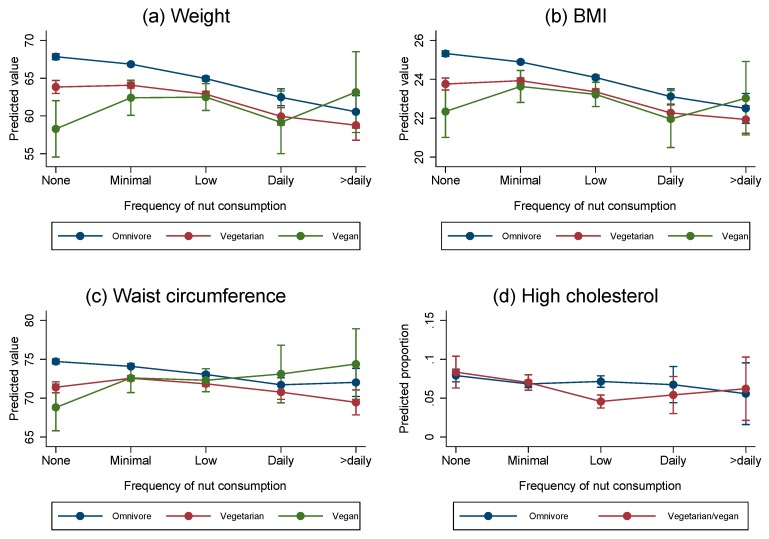
Association between frequency of nut consumption and predicted anthropometric and risk factors by dietary pattern. (**a**) Association between frequency of nut consumption and body weight by dietary pattern; (**b**) association between frequency of nut consumption and BMI by dietary pattern; (**c**) association between frequency of nut consumption and waist circumference by dietary pattern; (**d**) association between frequency of nut consumption and high blood cholesterol by dietary pattern.

For body weight, there was evidence of between diet group differences in trends for increasing nut frequency categories (interaction Wald *p* < 0.001) (as indicated by the non-parallel associations seen in Figure 1a, Table 6). Among omnivores, for each higher nut consumption category, the linear trend showed a 1.30 kg lower body weight (95% CI: 1.12, 1.48; *p* < 0.001) with a quadratic trend suggesting an additional negative trend (*p* = 0.003) and no evidence for higher order trends (*p* ≥ 0.220 for both). Among vegetarians, for those in each higher nut frequency category the linear trend shows a 0.73 kg lower mean body weight (95% CI: 0.46, 1.01; *p* < 0.001). However, among vegans, for each higher nut frequency category there was a non-statistically significant higher mean body weight by 0.87 kg (95% CI: −0.29, 2.04; *p* = 0.141). The linear negative trend for omnivores was statistically significantly greater than the trend in either vegetarians or vegans (both pairwise difference in trends *p* < 0.001) and there was also a statistically significant difference in the linear trend between vegetarians and vegans (*p* = 0.008). 

For BMI, there was again evidence of an overall difference in trends between diet groups (interaction Wald *p* < 0.001) (Figure 1b shows similar non-parallel slopes). Among omnivores, for each higher nut consumption category there was a 0.54 unit lower BMI (95% CI: 0.47, 0.60; *p* < 0.001), which was intensified in magnitude by a quadratic trend (*p* = 0.005). Among vegetarians, each higher nut consumption category was associated with a linear 0.3 unit lower BMI (95% CI: 0.20, 0.39; *p* < 0.001) further lowered by a statistically significant negative quadratic (*p* < 0.001), whereas for vegans, there was a non-statistically significant 0.07 unit higher BMI per category (95% CI: −0.34, 0.49; *p* = 0.727) and no evidence of a quadratic trend (*p* = 0.055). The linear trend for omnivores was statistically significantly more negative than that seen in vegetarians (difference in trends *p* < 0.001) and vegans (*p* = 0004), with no statistically significant difference between vegetarians and vegans (*p* = 0.087). The magnitude of the decreasing quadratic trend was greater among vegetarians than omnivores (*p* = 0.036). 

For waist circumference, there was evidence of a difference in trends (interaction Wald *p* < 0.001) and overall differences in linear (*p* < 0.001) and quadratic (*p* = 0.002) trends between diet groups (Figure 1c). Among omnivores, for each higher nut frequency category, there was a 0.69 cm unit lower mean waist circumference (95% CI: 0.54, 0.83; *p* < 0.001) with no evidence for higher order trends (all *p* ≥ 0.099). Among vegetarians each higher nut frequency category was associated with a non-statistically significant 0.13 cm lower mean waist circumference (95% CI: −0.36, 0.09; *p* = 0.241), whereas among vegans the trend was in the opposite direction with a statistically significant 0.96 cm higher mean waist circumference per category (95% CI: 0.01, 1.92; *p* = 0.047). The linear trend in omnivores was statistically significantly different from the trend observed among vegetarians (difference in trends *p* < 0.001) and vegans (*p* = 0.001) with a more negative quadratic trend evident among vegetarians compared to omnivores (*p* = 0.001). However, the quadratic trend (*p* < 0.001) in vegetarians dominated the linear trend, leading to lower waist circumferences for higher frequencies of nut consumption in this group. The difference between vegetarians and vegans in the linear trend was statistically significant (difference in trends *p* = 0.028), but not the quadratic trend (*p* = 0.704).

For the prevalence of having high blood cholesterol, the vegetarian and vegan groups were combined because of the lack of any respondents reporting high cholesterol for all nut frequency categories amongst the vegan group. There was evidence of an overall difference in trends between omnivores and a combined vegetarian/vegan group (interaction Wald *p* = 0.005) with the difference only evident for linear trends (Wald *p* = 0.004) and not for quadratic trends (*p* = 0.507), although there was a small but statistically significant cubic trend (*p* = 0.015) (Figure 1d). Among omnivores, for each greater nut frequency category, there was a non-statistically significant 4% lower odds of having high cholesterol (95% CI: 0.91, 1.02; *p* = 0.228). Among the combined vegetarian and vegan group, the odds were 19% lower for each higher category (95% CI: 0.73, 0.90; *p* < 0.001) but the cubic trend led to greater odds in the highest nut frequency categories.

There were also several overall differences of linear trends for a number of nutrients between diet groups which are presented briefly and without discussion of higher order trends below. For the percentage energy from total fat, saturated fat, monounsaturated fat, and polyunsaturated fat there was an overall difference between diet groups (all pairwise *p* < 0.001). Among omnivores, vegetarians, and vegans each higher nut frequency category was associated with a 1.4%, 1.9%, and 1.6% higher mean percentage of energy derived from total fat (all *p* < 0.001). The positive association for vegetarians was statistically significantly greater than for omnivores (*p* < 0.001).

For the percentage of energy from saturated fat, for each higher nut frequency category this was 0.1% and 0.2% higher for omnivores and vegetarians respectively (both *p* < 0.001). Conversely, for vegans, this was non-statistically significantly lower by 0.3% per nut consumption category (*p* = 0.097). The trend was statistically significantly different between all diet groups (all pairwise *p* ≤ 0.017).

For the percentage of energy from MUFA, for each higher nut frequency category was associated with 0.7%, 0.9%, and 0.7% higher means for omnivores, vegetarians, and vegans respectively (all *p* < 0.001). There was a statistically significant difference between omnivores and vegetarians only (*p* < 0.001).

For the percentage of energy from PUFA, for each higher nut frequency category, the means were 0.5%, 0.6%, and 0.9% greater for omnivores, vegetarians, and vegans respectively (all *p* < 0.001). There was evidence that the trend was different between all diet groups (all pairwise *p* < 0.001).

Amongst those with higher nut consumption categories, statistically significantly higher intakes were seen for protein among omnivores (4.5 g higher per category, *p* < 0.001), and vegetarians (3.8 g, *p* < 0.001). There was a non-statistically significantly greater mean by 1.8 g among vegans for each category (*p* = 0.187), with evidence of a difference between omnivores and vegan (*p* = 0.048).

For the percentage of energy from sugar there was also a statistically significant difference for linear trend between diet groups (Wald *p* = 0.006). For omnivores, vegetarians, and vegans there was a 0.4%, 0.7%, 1.0% higher percentage energy from sugar per higher category (all *p* ≤ 0.001). The difference between omnivores and vegetarians only was statistically significant (*p* = 0.006).

In terms of micronutrients, there were statistically significant differences in linear trends between diet groups for thiamine, vitamin B12, vitamin D, vitamin E, and calcium (all *p* < 0.001). For thiamine this association was positive for both omnivores (*p* < 0.001) and vegans (*p* = 0.023), but not for vegetarians (*p* = 0.307). For vitamin B12, each category higher nut frequency was associated with higher values among omnivores (*p* < 0.001), but with a statistically significant lower value among vegans (*p* = 0.010), and with no statistically significant difference among vegetarians (*p* = 0.411). There was evidence for different trends between all diet groups (all pairwise *p* ≤ 0.019).

For Vitamin D, each higher nut intake category was associated with higher values among omnivores (*p* < 0.001). There was no association for vegetarians and vegans. The trend was statistically significantly different between omnivores and vegetarians (*p* ≤ 0.001).

For vitamin E, for each greater nut frequency category, there was a 1.2 mg, 1.4 mg, and 1.9 mg higher mean for omnivores, vegetarians, and vegans respectively (all *p* < 0.001). The trend appeared to differ between the three diet groups (all pairwise *p* ≤ 0.013).

For calcium there was a positive association with nut consumption categories for both omnivores and vegetarians (both *p* < 0.001), but not for vegans (*p* = 0.874). There was evidence of different linear trends between omnivores and vegetarians (*p* = 0.027), and between omnivores and vegans (*p* = 0.019). 

## 4. Discussion

Examining the associations between nut intake and risk factors for chronic disease in this population with diverse dietary patterns has provided us with important information on the relationship between nut consumption and health. Higher nut consumption was associated with lower BMI, waist circumference, and prevalence of both high cholesterol and high blood pressure. In addition, higher nut consumption was associated with reduced prevalence of having had a heart attack, diabetes and gallstones. Greater nut intake was also associated with better diet quality overall. Predictors of nut consumption included younger age, greater education level, higher employment status, and more plant-based dietary categories. An understanding of these predictors can help target public health messages to increase regular nut consumption as part of a cardioprotective diet. 

A commonly reported barrier to nut consumption is the fear of weight gain because nuts are high in fat and energy [44]. However, we found nut consumption was inversely associated with body weight, BMI and waist circumference. These associations remained evident in models including physical activity and energy intake. These findings replicate those found by previous epidemiology studies which have reported that nut consumers are leaner than non-nut consumers [30,31,32,33,34]. Similarly, when nuts are added to the diet in intervention studies, weight gain is not apparent, or is at least less than predicted [34,35,36,37,38,39]. There have been several mechanisms for this finding discussed in the literature, including the high satiety effects of nuts [45,46], increased metabolic rate due to the high unsaturated fat content of nuts [47,48,49], and loss of metabolisable energy as faecal fat [50,51,52]. Given we found that higher nut consumption was associated with a leaner body composition, and lower abdominal fat, among a population with diverse dietary intakes, this adds to the evidence that fear of weight gain with increased nut consumption is unfounded. Therefore, public health messages should emphasise this point, and dispel such fears among the general population who are concerned that regular nut consumption could result in unwanted weight gain. 

There was evidence of a statistically significant linear trend where higher nut intake was associated with a lower prevalence of high blood cholesterol. This finding is supported by numerous intervention studies, which consistently report lower total and LDL cholesterol concentrations with greater nut consumption [23]. The prevalence of having had a heart attack was also lower amongst those with greater levels of nut consumption, a finding supported by numerous epidemiological studies, which have reported inverse associations between nut consumption and CVD [4,5,6,7,8,9,11]. One mechanism for these findings could be the higher intakes of unsaturated fat and lower intakes of saturated fat observed amongst those with greater nut intakes in this cohort. 

We found that with greater nut consumption, the prevalence of having high blood pressure was lower. While some studies are in agreement with this finding [24], not all studies have reported such an association [25]. Intervention studies have also produced equivocal findings [26,27]. Blood pressure is an important risk factor for stroke. In this study the prevalence of having had a stroke was not associated with nut intake. This is a finding congruent with a number of other studies [6,8,16].

Among this cohort of women, the prevalence of having been diagnosed with diabetes was lower amongst those with higher nut intakes. Previous research in this area has also produced mixed results, with one meta-analysis by Afshin et al. [53] reporting a small but significant reduction in the incidence of type 2 diabetes with higher nut consumption, whereas a further three meta-analyses have reported no association [8,15,16]. The inconsistent results could have resulted from lack of adjustment for BMI by Afshin et al. [53]. In our study, although further adjustment for BMI attenuated the relationship between nut intake and the incidence of diabetes, the association remained statistically significant, and it is important to consider that BMI could both be a confounder of any such association and also be on the causal pathway between nut consumption and diabetes. Possible mechanisms for a reduction in the risk of developing type 2 diabetes from greater nut intakes are the higher intakes of monounsaturated and polyunsaturated fats, which were evident in this cohort, using both absolute amounts and as a percentage of total energy, for those with greater nut intakes. Higher intakes of these unsaturated fats are associated with improvements in insulin sensitivity [54]. However, given the inconsistent findings of research in this area to date, future carefully designed studies are required to address the possible relationship between nut intake and the development of type 2 diabetes.

Diet quality was better amongst those with higher nut intakes. As mentioned above, nut intake was associated with a more cardioprotective fat intake, namely, a higher percentage of energy from cis-unsaturated fats, with a lower proportion from saturated fat. Greater levels of nut consumption were also associated with a more nutrient-dense diet in terms of vitamins and minerals (except for vitamin A, C and B12), and also higher intakes of fibre, and lower intakes of sugar. The better nutrient profile observed among nut consumers is consistent with both previous epidemiologic [19,20,21,55,56] and intervention trials [39,57,58]. In fact, the results are remarkably similar to a NZ cross-sectional study using a 24-h recall, which showed higher intakes of unsaturated fats, lower intakes of saturated fat, higher intakes in most nutrients except for vitamin A and B12 [55]. This consistency is reassuring given that the studies used different dietary assessment techniques and were conducted using data from different populations.

In order to promote regular nut consumption, gaining some information on the factors associated with nut intake is important. Predictors of greater intake in this UK cohort included older age, more skilled job categories, higher levels of education, and more plant-based dietary patterns. Age has been associated with nut consumption in other epidemiological studies [17,21], although differences in age classification make comparisons difficult. In this cohort, nut consumption was negatively associated with age but in a US study, the percentage of those consuming nuts was higher in those aged over 51 years compared to those aged 19–51 years [21] and in a NZ sample, nut consumption was significantly higher among those aged 31–70 years compared those aged 18–30 years [17]. Differences in findings are likely to be due to differences in the age range studied. The UK cohort included participants aged 35–70 years, whereas those in the NZ and USA studies had lower age limits of 18 and 19 years respectively. 

Higher education and occupational status were associated with a higher likelihood of consuming nuts. Education has also been positively associated with nut intake in NZ, Europe and the USA [17,21,59]. Education level and occupation can, with caution, be used as a rough proxy for socio-economic status (SES). Studies in NZ and the USA have reported a higher prevalence of nut intake among those with a higher SES [17]. Cost has been reported as a barrier to regular nut consumption [44]. Therefore public health initiatives could consider promoting more affordable nuts such as peanuts, which have been shown to improve health [11,35,56,60,61,62,63]. 

Those who were vegetarian or vegans were unsurprisingly more likely to consume nuts compared to those who reported eating meat, a finding supported by previous research [18,22]. Vegetarians, and in particular, vegans, are at higher risk of certain nutrient deficiencies compared to omnivores [64]. It is therefore reassuring that they consume more nuts, given that nuts are nutrient dense, and important sources of protein, unsaturated fats and a number of vitamins and minerals including iron and zinc [65]. 

A novel finding of this study is that the benefits of higher levels of nut consumption appear to be greater overall amongst omnivores than vegetarians and vegans, with vegans showing the least benefits in terms of anthropometry outcomes with higher nut intakes and some suggestions of potentially worse outcomes for the highest intake category (60 g per day and above) although these should be interpreted with caution due to the small number of participants in this group. This overall pattern may reflect generally healthier diets amongst non-omnivores with higher levels of nut consumption being associated with smaller differences between the dietary groups. This differs from the Seventh-Day Adventist results where statistically significant associations were observed for both vegetarians and non-vegetarians (vegans were not independently examined), and while that study did not investigate effect modification by dietary pattern, the protective gradient appeared steeper in vegetarians [22]. If our results are confirmed, then where appropriate, public health messages encouraging greater levels of nut consumption could be specifically targeted at omnivores, for example by encouraging incorporating nuts into meat dishes as well as consuming them as a snack food. As this is the largest dietary group in most western countries, and as the benefits from increasing intakes may be greater within this group than for non-omnivores, such targeted interventions could maximise the population–level impact from uptake of the intervention messages. The pattern of worsening anthropometry measures amongst vegans with the highest levels of nut intake (60 g per day and above) or having high cholesterol amongst vegetarians and vegans for the same high intake is also novel although these differences between 60 g per day and no nuts for these diet groups was only statistically significant for waist circumference (*p* = 0.045). Further research is needed as to whether this is a marker of particular vegan or vegetarian/vegan dietary patterns or whether this finding is spurious. While consumption of nuts has been found to be associated with lower BMI in general population surveys, such high intakes of nuts may not have the same association amongst vegans. It is worth noting that vegans in this highest intake category did not appear to be consuming meaningfully more nuts per day (88 g) than omnivores (85 g) or vegetarians (96 g) although these are all well above common recommendations for nut intakes.

There are several limitations which should be considered when interpreting the results of this study. Firstly, this was a cross-sectional analysis and so causal inference cannot be drawn, although consistency between the data and plausible causal models can be examined. The preferable risk factor profiles, and disease incidences observed amongst those with higher nut intakes might be due to the addition of nutrient-dense nuts to the diet; however, an alternative explanation could be that nut consumers are more health conscious. Thus, high nut consumption could also be a marker of a healthier lifestyle and the observed associations could be the result of residual confounding by health consciousness and associated health behaviours, despite the wide range of potential confounders included in the adjusted analyses. We have not, for example, adjusted for physical activity or stress and these could plausibly confound these associations. In general, the UKWCS was comprised of participants who appeared health conscious, with relatively low rates of smoking and low BMI. Additionally, this cohort included only females, and so results cannot be directly extrapolated to males. Nut intake was assessed by FFQ, which tends to over-estimate the consumption of certain foods [66]. However, the FFQ used has been validated against a semi-weighed four-day food dairy and biomarkers of intake [41]. In addition, a strength of this study is diverse nature of the population in terms of dietary patterns resulting in the wider range in nut intake than seen in other research, which reduces estimation error [67,68].

## 5. Conclusions

In conclusion, higher nut intakes in this cohort, which comprised participants with diverse dietary patterns, were associated with a lower prevalence of having diabetes, gallstones, and a heart attack. Better risk profiles from lower BMI, abdominal fat, blood cholesterol and blood pressure could account for these observed reductions. Further, the better diet quality observed amongst those with higher nut intakes, in particular with the type of fat, are likely to contribute to the protective effects of regular nut consumption. Predictors of nut intakes included age, education level and employment status, which are relatively consistent with other studies. The associations of more positive markers of health with higher levels of nut intake appear to be greatest for omnivores, followed by vegetarians, and least, and negatively associated in one case, for vegans. These predictors should be used to guide future health messages aimed at increasing regular nut consumption, especially amongst omnivores.

## Figures and Tables

**Table 1:** Characteristics of study participants by nut consumption.

Demographic Variable	All Participants * (*n* = 34,831)	Non-Nut Consumers (*n* = 5631)	Nut Consumers (*n* = 29,200)
Age (years) ^a^	52.2 (9.3)	55.5 (9.6)	51.6 (9.1)
Height (cm) ^a^	163.7 (6.8)	163.1 (6.8)	163.8 (6.7)
Weight (kg) ^a^	65.6 (11.9)	67.4 (13.2)	65.3 (11.6)
BMI (kg/m^2^) ^a^	24.5 (4.3)	25.3 (4.7)	24.3 (4.1)
Waist circumference (cm) ^a^	73.6 (9.3)	75.0 (10.3)	73.3 (9.0)
Physical activity ^b^			
None	42.2 (13,934)	52.9 (2769)	40.2 (11,165)
<75 min/week	20.0 (6606)	14.1 (738)	21.1 (5868)
75 min/week or more	37.7 (12,448)	33.0 (1725)	38.6 (10,723)
Ethnicity ^b^			
White	96.0 (33,432)	95.9 (5400)	96.0 (28,032)
Asia	0.5 (188)	0.1 (8)	0.6 (180)
Black	0.1 (50)	0.1 (6)	0.2 (44)
Other	3.3 (1161)	3.9 (217)	3.2 (944)
Employment ^b^			
Professional	24.7 (8590)	18.2 (1024)	25.9 (7566)
Managerial, technical, admin	28.7 (9994)	23.9 (1346)	29.6 (8648)
Clerical/Skilled	41.2 (14,338)	49.0 (2760)	39.7 (11,578)
Manual	1.0 (344)	1.6 (89)	0.9 (255)
Other/missing/no job	4.5 (1565)	7.3 (412)	4.0 (1153)
Highest educational achievement ^b^			
No qualifications	16.8 (5347)	30.2 (1507)	14.3 (3840)
O-level (16 years)	31.2 (9940)	33.8 (1688)	30.7 (8252)
A-level (18 years)	24.7 (7859)	20.2 (1009)	25.5 (6850)
Degree	27.4 (8718)	15.9 (794)	29.5 (7924)
Dietary pattern ^b^			
Omnivore	72.1 (25,116)	83.1 (4679)	70.0 (20,437)
Vegetarian	26.6 (9280)	16.1 (905)	28.7 (8375)
Vegan	1.3 (435)	0.8 (47)	1.3 (388)
Energy intake (kJ/day) ^a^	9817 (2986)	9029 (2954)	9969 (2986)
Alcohol			
consume any alcohol ^b^	89.6 (29,848)	80.4 (4302)	91.3 (25,546)
g/day among alcohol consumers ^c^	6.7 (11.8)	4.1 (10.1)	7.0 (12.1)
Smoking status ^b^			
Never smoked	57.9 (19,535)	58.8 (3186)	57.7 (16,349)
Current smoker	11.1 (3739)	12.1 (657)	10.9 (3082)
Former smoker	31.1 (10,486)	29.1 (1580)	31.4 (8906)
Menopausal status ^b^			
Premenopausal	47.6 (15,859)	33.0 (1768)	50.4 (14,091)
Postmenopausal	52.4 (17,462)	67.0 (3586)	49.6 (13,876)
Current HRT use (%) ^b^	23.2 (7539)	23.1 (1195)	23.2 (6344)
Current OCP use (%) ^b^	3.9 (1294)	3.2 (166)	4.1 (1128)
Age of menarche (year) ^a^	12.8 (1.6)	12.8 (1.7)	12.8 (1.6)
Parity ^b^			
no children	13.6 (4258)	13.3 (667)	13.7 (3591)
1–2 children	65.9 (17,775)	55.6 (2790)	57.2 (14,985)
3–4 children	27.1 (8449)	27.9 (1399)	26.9 (7050)
≥5 children	2.3 (724)	3.2 (162)	2.2 (562)

* Included only participants with data on nut consumption (*n* = 34,831); ^a^ values are presented as means (standard deviations); ^b^ values are presented as % (number); ^c^ values are presented as medians (IQR); abbreviations: BMI, body mass index; HRT, hormone replacement therapy; OCP, oral contraceptive pill.

**Table 2:** Predictors of nut consumption *.

Demographic Variable	Total Nut Consumption					Unadjusted OR ^†^	Unadjusted *p*-Value	Adjusted OR ^†,‡^	Adjusted *p*-Value ^‡^
	No Nuts (*n* = 5631)	<Once per Week (*n* = 17,992)	2–6 Times per Week (*n* = 9887)	Daily (*n* = 995)	>Daily (*n* = 326)				
Age (years) OR per 5 years ^1^	55.5 (55.3, 55.8)	51.8 (51.7, 52.0)	51.0 (50.8, 51.2)	53.3 (52.7, 53.9)	54.1 (53.1, 55.2)		<0.001		<0.001
OR for 50 vs. 40						0.84 (0.83, 0.85)		0.95 (0.93, 0.97)	
OR for 60 vs. 40						0.65 (0.63, 0.67)		0.88 (0.84, 0.92)	
OR for 70 vs. 40						0.45 (0.42, 0.48)		0.79 (0.73, 0.86)	
Physical activity ^2^									
None	52.9 (2769)	42.5 (7262)	36.4 (3436)	36.6 (348)	38.8 (119)	1.00 ^a^	<0.001	1.00 ^a^	<0.001
<75 min/week	14.1 (738)	20.8 (3543)	21.8 (2054)	21.7 (206)	21.2 (65)	1.54 (1.45, 1.62) ^b^		1.31 (1.24, 1.40) ^b^	
75 min/week or more	33.0 (1725)	36.7 (6264)	41.8 (3940)	41.7 (396)	40.1 (123)	1.47 (1.41, 1.54) ^b^		1.29 (1.23, 1.36) ^b^	
Employment ^2^							<0.001		<0.001
Professional	18.2 (1024)	24.3 (4370)	28.3 (2794)	32.5 (323)	24.2 (79)	1.00 ^a^		1.00 ^a^	
Managerial, technical, admin	23.9 (1346)	28.5 (5133)	31.7 (3138)	29.0 (289)	27.0 (88)	0.90 (0.85, 0.95) ^b^		1.12 (1.05, 1.19) ^b^	
Clerical/Skilled	49.0 (2760)	42.1 (7580)	35.6 (3514)	34.4 (342)	43.6 (142)	0.63 (0.60, 0.66) ^c^		1.01 (0.94, 1.08) ^a^	
Manual	1.6 (89)	1.0 (183)	0.7 (67)	0.2 (2)	0.9 (3)	0.42 (0.35, 0.52) ^d^		0.87 (0.68, 1.10) ^a,b^	
other/missing/no job	7.3 (412)	4.0 (726)	3.8 (374)	3.9 (39)	14 (4.3%)	0.51 (0.46, 0.56) ^d^		0.91 (0.80, 1.04) ^a^	
Highest educational achievement (%) ^2^							<0.001		<0.001
no qualifications	30.2 (1507)	16.1 (2656)	11.0 (1008)	13.0 (120)	18.4 (56)	1.00 ^a^		1.00 ^a^	
O-level (16 years)	33.8 (1688)	32.8 (5404)	27.5 (2529)	25.4 (234)	28.0 (85)	1.66 (1.55, 1.77) ^b^		1.41 (1.31, 1.52) ^b^	
A-level (18 years)	20.2 (1009)	24.9 (4092)	26.7 (2456)	24.5 (226)	25.0 (76)	2.24 (2.09, 2.39) ^c^		1.87 (1.73, 2.03) ^c^	
Degree	15.9 (794)	26.2 (4302)	34.8 (3194)	37.0 (341)	28.6 (87)	2.98 (2.78, 3.18) ^d^		2.26 (2.07, 2.46) ^d^	
Dietary status ^2^							<0.001		<0.001
Omnivore	83.1 (4679)	77.4 (13,931)	59.3 (5861)	50.9 (506)	42.6 (139)	1.00 ^a^		1.00 ^a^	
Vegetarian	16.1 (905)	21.9 (3932)	38.7 (3823)	45.7 (455)	50.6 (165)	2.51 (2.40, 2.63) ^b^		2.23 (2.12, 2.35) ^b^	
Vegan	0.8 (47)	0.7 (12)	2.1 (203)	3.4 (34)	6.8 (22)	4.06 (3.36, 4.89) ^c^		3.76 (3.05, 4.64) ^c^	
Energy intake (kJ/day) ^1^	9029 (8952, 9107)	9474 (9434, 9514)	10,565 (10,505, 10,642)	11,819 (11,610, 12,028)	13,596 (13,170, 14,022)		<0.001		
OR for 4 MJ vs. 10 MJ						0.31 (0.29, 0.32)			
OR for 6 MJ vs. 10 MJ						0.49 (0.47, 0.50)			
OR for 8 MJ vs. 10 MJ						0.71 (0.70, 0.72)			
OR for 12 MJ vs. 10 MJ						1.36 (1.34, 1.38)			
OR for 14 MJ vs. 10 MJ						1.80 (1.75, 1.85)			
OR for 16 MJ vs. 10 MJ						2.34 (2.25, 2.43)			
Smoking status ^2^									
Never smoked	58.8 (3186)	57.9 (10,132)	57.2 (5482)	57.3 (545)	61.5 (190)	1.00 ^a^	<0.001	1.00 ^a^	<0.001
Current smoker	12.1 (657)	11.0 (1928)	10.8 (1034)	9.8 (93)	8.7 (27)	0.94 (0.88, 1.00) ^a^		0.98 (0.91, 1.05) ^a^	
Former smoker	29.1 (1580)	31.0 (5430)	32.0 (3071)	32.9 (313)	29.8 (92)	1.07 (1.02, 1.12) ^b^		1.07 (1.02, 1.12) ^b^	

* Included participants with data on nut consumption (*n* = 34,831); ^1^ Values are presented as means (95% CI); ^2^ values are presented as % (number); ^†^ calculated using ordinal logistic regression with fractional polynomials used for continuous variables; ^‡^ adjusted for all other variables in the table except for energy intake; MJ, Megajoule; OR, Odds ratio. Odds ratios with different superscript letters (e.g., ^a^ and ^b^) are significantly different.

**Table 3:** Anthropometric measures by nut consumption category.

Anthropometry Variable	Total Nut Consumption						*p* for Linear Trend Unadjusted ^†^	*p* for Linear Trend Adjusted ^†,‡^	*p* for Linear trend Further Adjusted for Energy Intake ^†,‡^
	All Participants (*n* = 34,831)	No Nuts (*n* = 5631)	<Once per Week (*n* = 17,992)	2–6 Times per Week (*n* = 9887)	Daily (*n* = 995)	>Daily (*n* = 326)			
Weight (kg)	65.6 (65.5, 65.7)	67.4 (67.1, 67.8)	66.2 (66.0, 66.4)	64.0 (63.8, 64.3)	61.7 (63.8, 64.3)	60.6 (59.5, 61.7)	<0.001 ^§^	<0.001 ^§^	<0.001 ^§^
BMI (kg/m^2^)	24.5 (24.4, 24.5)	25.3 (25.2, 25.4)	24.7 (24.7, 24.8)	23.7 (23.6, 23.9)	22.8 (22.6, 23.1)	22.5 (22.2, 22.9)	<0.001 ^§^	<0.001 ^§^	<0.001 ^§^
Waist circumference (cm)	73.6 (73.5, 73.7)	75.0 (74.7, 75.3)	73.8 (73.6, 74.0)	72.5 (72.3, 72.7)	72.2 (71.6, 72.8)	72.1 (71.1, 73.2)	<0.001 ^§^	<0.001	<0.001 ^§^

Values are presented as means (95% CI); ^†^ calculated using orthogonal polynomials; ^‡^ adjusted for age, vigorous physical activity category, dietary pattern, job category, and smoking status; ^§^ there was statistical significant evidence for a quadratic or higher order trend.

**Table 4:** Odds ratio (95%CI) for chronic disease and risk factors for chronic disease by nut consumption.

Disease/Risk Factor Variable	Total Nut Consumption					*p* for Linear Trend ^†^
	No Nuts (*n* = 5631)	<Once per Week (*n* = 17,992)	2–6 Times per Week (*n* = 9887)	Daily (*n* = 995)	>Daily (*n* = 326)	
High Blood Pressure						
Cases	1190	2945	1329	144	44	
Unadjusted	1.00	0.70 (0.65, 0.75)	0.55 (0.51, 0.60)	0.60 (0.5, 0.73)	0.54 (0.40, 0.76)	<0.001
Adjusted for age only	1.00	0.86 (0.80, 0.93)	0.71 (0.65, 0.78)	0.68 (0.56, 0.83)	0.57 (0.41, 0.80)	<0.001
Adjusted ^‡^	1.00	0.85 (0.78, 0.93)	0.75 (0.67, 0.83)	0.72 (0.58, 0.90)	0.69 (0.48, 0.98)	<0.001
Further adjusted for BMI	1.00	0.86 (0.78, 0.93)	0.82 (0.74, 0.91)	0.90 (0.72, 1.12)	0.92 (0.63, 1.34)	0.010
High cholesterol						
Cases	547	1261	564	58	20	
Unadjusted ^‡^	1.00	0.66 (0.60, 0.74)	0.53 (0.47, 0.60)	0.54 (0.41, 0.72)	0.57 (0.36, 0.90)	<0.001
Adjusted for age only	1.00	0.87 (0.78, 0.97)	0.73 (0.64, 0.83)	0.63 (0.47, 0.83)	0.62 (0.38, 0.99)	<0.001
Adjusted ^‡^	1.00	0.84 (0.74, 0.95)	0.75 (0.65, 0.87)	0.70 (0.51, 0.96)	0.67 (0.40, 1.14)	<0.001
Further adjusted for BMI	1.00	0.85 (0.75, 0.96)	0.78 (0.67, 0.91)	0.78 (0.57, 1.08)	0.76 (0.44, 1.32)	0.004
Heart Attack						
Cases	151	212	101	17	2	
Unadjusted	1.00	0.41 (0.33, 0.51)	0.35 (0.27, 0.46)	0.59 (0.36, 0.99)	0.21 (0.05, 0.86)	<0.001
Adjusted for age only	1.00	0.59 (0.47, 0.73)	0.55 (0.42, 0.72)	0.73 (0.44, 1.22)	0.23 (0.06, 0.94)	<0.001
Adjusted ^‡^	1.00	0.56 (0.44, 0.72)	0.51 (0.37, 0.69)	0.70 (0.38, 1.26)	0.28 (0.07, 1.15)	<0.001
Further adjusted for BMI	1.00	0.56 (0.44, 0.72)	0.55 (0.40, 0.75)	0.82 (0.45, 1.49)	0.36 (0.09, 1.51)	0.011
Angina						
Cases	206	314	144	20	9	
Unadjusted	1.00	0.44 (0.37, 0.53)	0.37 (0.30, 0.46)	0.51 (0.32, 0.81)	0.70 (0.25, 1.37)	<0.001
Adjusted for age only	1.00	0.65 (0.54, 0.79)	0.60 (0.48, 0.74)	0.63 (0.39, 1.01)	0.78 (0.39, 1.55)	<0.001
Adjusted ^‡^	1.00	0.64 (0.51, 0.79)	0.63 (0.48, 0.82)	0.64 (0.36, 1.12)	0.85 (0.38, 1.88)	0.016
Further adjusted for BMI	1.00	0.65 (0.52, 0.82)	0.67 (0.51, 0.88)	0.74 (0.41, 1.30)	0.95 (0.40, 2.22)	0.092 ^§^
Stroke						
Cases	79	109	51	10	4	
Unadjusted	1.00	0.41 (0.30, 0.54)	0.34 (0.24, 0.49)	0.67 (0.35, 1.30)	0.82 (0.30, 2.25)	<0.001
Adjusted for age only	1.00	0.55 (0.41, 0.74)	0.51 (0.35, 0.73)	0.79 (0.41, 1.54)	0.89 (0.32, 2.48)	0.023
Adjusted ^‡^	1.00	0.62 (0.44, 0.87)	0.54 (0.36, 0.83)	0.89 (0.42, 1.91)	1.31 (0.46, 3.71)	0.115 ^§^
Further adjusted for BMI	1.00	0.61 (0.43, 0.86)	0.56 (0.36, 0.86)	0.94 (0.44, 2.02)	1.48 (0.52, 4.21)	0.201 ^§^
Diabetes						
Cases	197	297	119	12	5	
Unadjusted	1.00	0.44 (0.37, 0.53)	0.32 (0.25, 0.40)	0.32 (0.18, 0.57)	0.40 (0.16, 0.99)	<0.001
Adjusted for age only	1.00	0.54 (0.45, 0.66)	0.41 (0.32, 0.52)	0.35 (0.20, 0.65)	0.44 (0.18, 1.07)	<0.001
Adjusted ^‡^	1.00	0.58 (0.47, 0.72)	0.46 (0.35, 0.60)	0.57 (0.31, 1.04)	0.54 (0.20, 1.50)	<0.001
Further adjusted for BMI	1.00	0.61 (0.48, 0.76)	0.54 (0.40, 0.72)	0.79 (0.43, 1.44)	0.89 (0.32, 2.46)	0.008
Gallstones						
Cases	440	931	391	48	13	
Unadjusted	1.00	0.61 (0.54, 0.69)	0.46 (0.40, 0.53)	0.56 (0.41, 0.76)	0.46 (0.26, 0.80)	<0.001
Adjusted for age only	1.00	0.73 (0.65, 0.83)	0.58 (0.50, 0.66)	0.59 (0.43, 0.81)	0.49 (0.28, 0.86)	<0.001
Adjusted ^‡^	1.00	0.77 (0.67, 0.88)	0.64 (0.55, 0.76)	0.73 (0.52, 1.03)	0.46 (0.24, 0.92)	<0.001
Further adjusted for BMI	1.00	0.78 (0.68, 0.90)	0.70 (0.59, 0.83)	0.85 (0.60, 1.22)	0.63 (0.32, 1.25)	0.002
Polyps						
Cases	85	172	93	4	4	
Unadjusted	1.00	0.60 (0.46, 0.77)	0.58 (0.43, 0.78)	0.25 (0.09, 0.67)	0.76 (0.28, 2.07)	0.001
Adjusted for age only	1.00	0.78 (0.60, 1.02)	0.80 (0.59, 1.09)	0.29 (0.10, 0.78)	0.62 (0.19, 1.97)	0.028
Adjusted ^‡^	1.00	0.80 (0.58, 1.09)	0.83 (0.58, 1.19)	0.31 (0.10, 0.99)	0.85 (0.26, 2.79)	0.119
Further adjusted for BMI	1.00	0.81 (l.59, 1.11)	0.85 (0.59, 1.24)	0.34 (0.11, 1.09)	1.01 (0.31, 3.33)	0.206
Cancer						
Cases	463	1175	671	72	20	
Unadjusted	1.00	0.74 (0.66, 0.83)	0.77 (0.68, 0.87)	0.81 (0.63, 1.05)	0.68 (0.43, 1.09)	0.003
Adjusted for age only	1.00	0.89 (0.80, 1.00)	0.98 (0.86, 1.11)	0.91 (0.70, 1.19)	0.66 (0.40, 1.07)	0.632 ^§^
Adjusted ^‡^	1.00	1.01 (0.88, 1.15)	1.13 (0.97, 1.31)	0.95 (0.70, 1.29)	0.78 (0.46, 1.34)	0.366
Further adjusted for BMI	1.00	1.01 (0.88, 1.16)	1.15 (0.98, 1.33)	0.94 (0.68, 1.28)	0.84 (0.49, 1.44)	0.267

^†^ Calculated using orthogonal polynomials; ^‡^ adjusted for age, vigorous physical activity category, dietary pattern, job category, and smoking status; ^§^ there was statistical significant evidence for a quadratic or higher order trend.

**Table 5:** Nutrient intakes by nut consumption category.

Nutrient	Total Nut Consumption						*p* for Linear Trend Unadjusted ^†,‡^	*p* for Linear Trend Adjusted ^†,‡^	*p* for Linear Trend Further Adjusted for Energy Intake ^†,‡^
	All Participants (*n* = 34,831)	No Nuts (*n* = 5631)	<Once per Week (*n* = 17,992)	2–6 Times per Week (*n* = 9887)	Daily (*n* = 995)	>Daily (*n* = 326)			
Total energy (kJ)	9817 (9786, 9849)	9029 (8952, 9107)	9474 (9434, 9514)	10,565 (10,504, 10,624)	11,819 (11,610, 12,028)	13,596 (13,169, 14,023)	<0.001 ^§^	<0.001^§^	
Total fat (g)	84.7 (84.4, 85.0)	73.3 (72.5, 74.1)	80.3 (79.9, 80.8)	94.4 (93.8, 95.1)	112.3 (110.0, 114.6)	142.7 (137.2, 148.2)	<0.001 ^§^	<0.001 ^§^	
%TE	32.4 (32.4, 32.5)	30.5 (30.3, 30.6)	32.0 (31.9, 32.1)	33.7 (33.6, 33.8)	35.9 (35.5, 36.2)	39.5 (38.8, 40.3)	<0.001 ^§^	<0.001 ^§^	
Saturated fat (g)	29.3 (29.2, 29.4)	26.5 (26.1, 26.8)	28.5 (28.3, 28.7)	31.7 (31.4, 31.9)	33.8 (32.8, 34.7)	37.4 (35.6, 39.2)	<0.001 ^§^	<0.001 ^§^	
%TE	11.1 (11.1, 11.1)	10.9 (10.8, 11.0)	11.2 (11.2, 11.2)	11.2 (11.1, 11.2)	10.6 (10.4, 10.8)	10.3 (9.9, 10.6)	0.938 ^§^	<0.001 ^§^	
Monounsaturated fat (g)	27.7 (27.6, 27.8)	23.4 (23.1, 23.6)	26.0 (25.9, 26.1)	31.2 (31.0, 31.4)	39.0 (38.2, 39.7)	52.9 (50.7, 55.2)	<0.001 ^§^	<0.001 ^§^	
%TE	10.5 (10.5, 10.6)	9.7 (9.6, 9.7)	10.3 (10.3, 10.3)	11.1 (11.1, 11.2)	12.5 (12.3, 12.6)	14.7 (14.3, 15.1)	<0.001 ^§^	<0.001 ^§^	
Polyunsaturated fat (g)	16.3 (16.2, 16.4)	13.4 (13.2, 13.6)	15.0 (14.9, 15.1)	18.8 (18.6, 18.9)	25.0 (24.5, 25.5)	36.2 (34.6, 37.8)	<0.001 ^§^	<0.001 ^§^	
%TE	6.2 (6.2, 6.2)	5.6 (5.5, 5.6)	6.0 (6.0, 6.0)	6.7 (6.7, 6.8)	8.1 (8.0, 8.2)	10.1 (9.8, 10.4)	<0.001 ^§^	<0.001 ^§^	
Protein (g)	89.3 (89.0, 89.6)	87.5 (86.8, 88.2)	87.7 (87.3, 88.1)	91.5 (91.0, 92.1)	98.1 (96.2, 100.0)	110.1 (106.4, 113.9)	<0.001 ^§^	<0.001 ^§^	
%TE	15.1 (15.0, 15.1)	16.1 (16.0, 16.2)	15.3 (15.2, 15.3)	14.3 (14.3, 14.4)	13.8 (13.7, 13.9)	13.5 (13.3, 13.8)	<0.001 ^§^	<0.001 ^§^	
Carbohydrate (g)	313 (312, 314)	297 (294, 300)	303 (302, 305)	322 (330, 335)	365 (358, 372)	394 (379, 409)	<0.001 ^§^	<0.001 ^§^	
%TE	52.6 (52.6, 52.7)	54.2 (54.0, 54.4)	52.7 (52.6, 52.8)	51.9 (51.8, 52.1)	51.0 (50.6, 51.4)	48.2 (47.3, 49.1)	<0.001 ^§^	<0.001 ^§^	
Sugar (g)	149 (148, 149)	142 (140, 144)	143 (142, 144)	157 (156, 159)	180 (176, 185)	199 (189, 208)	<0.001 ^§^	<0.001 ^§^	
%TE	25.2 (25.2, 25.3)	26.2 (26.0, 26.4)	25.1 (25.1, 25.2)	24.8 (24.7, 24.9)	25.5 (25.1, 25.9)	24.3 (23.5, 25.0)	<0.001 ^§^	<0.001 ^§^	
Fibre (g)	25.6 (25.5, 25.7)	23.6 (23.3, 23.9)	24.3 (24.1, 24.4)	28.0 (27.8, 28.2)	33.1 (32.3, 33.8)	39.5 (37.7, 41.3)	<0.001 ^§^	<0.001 ^§^	0.002 ^§^
Vitamin A (μg)	1243 (1236, 1249)	1175 (1158, 1192)	1219 (1210, 1227)	1303 (1291, 1314)	1390 (1349, 1431)	1477 (1382, 1572)	<0.001 ^§^	<0.001 ^§^	0.340 ^§^
Thiamin (mg)	3.13 (3.10, 3.15)	2.79 (2.72, 2.85)	2.94 (2.90, 2.97)	3.54 (3.48, 3.59)	3.94 (3.76, 4.13)	4.65 (4.28, 5.03)	<0.001 ^§^	<0.001 ^§^	<0.001
Riboflavin (mg)	2.52 (2.51, 2.53)	2.48 (2.46, 2.50)	2.47 (2.46, 2.48)	2.58 (2.56, 2.60)	2.75 (2.69, 2.81)	2.94 (2.81, 3.06)	<0.001 ^§^	<0.001 ^§^	<0.001 ^§^
Vitamin B6 (mg)	2.82 (2.81, 2.83)	2.77 (2.74, 2.79)	2.75 (2.74, 2.76)	2.92 (2.90, 2.94)	3.19 (3.13, 3.25)	3.62 (3.48, 3.75)	<0.001 ^§^	<0.001 ^§^	<0.001 ^§^
Vitamin B12 (μg)	5.69 (5.66, 5.72)	5.93 (5.85, 6.01)	5.80 (5.75, 5.84)	5.44 (5.37, 5.50)	5.21 (4.99, 5.43)	4.76 (4.38, 5.15)	<0.001 ^§^	<0.001 ^§^	<0.001 ^§^
Folate (μg)	402 (401, 403)	384 (381, 388)	388 (386, 389)	426 (423, 429)	476 (466, 485)	550 (527, 573)	<0.001 ^§^	<0.001 ^§^	<0.001 ^§^
Vitamin C (mg)	171 (170, 172)	160 (158, 163)	164 (163, 165)	185 (184, 187)	206 (200, 212)	230 (213, 246)	<0.001 ^§^	<0.001 ^§^	0.664 ^§^
Vitamin E (mg)	9.71 (9.66, 9.75)	8.19 (8.09, 8.29)	9.01 (8.96, 9.07)	11.09 (11.01, 11.18)	14.14 (13.84, 14.44)	18.74 (17.97, 19.52)	<0.001 ^§^	<0.001 ^§^	<0.001 ^§^
Calcium (mg)	1137 (1133, 1141)	1099 (1089, 1109)	1114 (1109, 1120)	1182 (1174, 1190)	1252 (1225, 1279)	1367 (1313, 1421)	<0.001 ^§^	<0.001 ^§^	<0.001 ^§^
Iron (mg)	18.8 (18.8, 18.9)	17.8 (17.5, 18.0)	18.2 (18.1, 18.3)	20.0 (19.9, 20.2)	22.7 (22.1, 23.2)	25.7 (24.6, 26.9)	<0.001 ^§^	<0.001 ^§^	<0.001
Zinc (mg)	11.5 (11.4, 11.5)	11.0 (10.9, 11.1)	11.2 (11.1, 11.2)	11.9 (11.9, 12.0)	13.3 (13.1, 13.6)	15.6 (15.1, 16.2)	<0.001 ^§^	<0.001 ^§^	<0.001 ^§^

Values are presented as means (95% CI); ^†^ calculated using orthogonal polynomials; ^‡^ adjusted for age, vigorous physical activity category, dietary pattern, job category, and smoking status; values with different superscript letters are significantly different in the adjusted model; ^§^ there was statistically significant evidence for a quadratic or higher order trend; TE, total energy.

**Table 6:** Effect modification of anthropometric and risk factor associations by dietary pattern.

Anthropometry/Risk Factor Variable	Linear Trend *				Quadratic Trend after Accounting for Linear Trend *				All Trends Combined *
	Omnivores	Vegetarians	Vegans	*p*-Value for between Group Difference	Omnivores	Vegetarians	Vegans	*p*-Value for between Group Difference	*p*-Value for between Group Difference
Weight (kg)	−1.30 (−1.48, −1.12)	−0.73 (−1.01, −0.46)	0.87 (−0.29, 2.04)	<0.001	−0.28 (−0.46, −0.09)	−0.53 (−0.79, −0.27)	−1.08 (−2.20, 0.05)	0.133	<0.001
BMI (kg/m^2^)	−0.54 (−0.60, −0.47)	−0.30 (−0.39, −0.20)	0.07 (−0.34, 0.49)	<0.001	−0.09 (−0.16, −0.03)	−0.21 (−0.31, −0.12)	−0.39 (−0.79, 0.01)	0.050	<0.001
Waist circumference (cm)	−0.69 (−0.83, −0.54)	−0.13 (−0.36, 0.09)	0.96 (0.01, 1.92)	<0.001	−0.04 (−0.19, 0.11)	−0.50 (−0.72, −0.29)	−0.68 (−1.60, 0.24)	0.002	<0.001
High cholesterol ^†^	0.96 (0.91, 1.02)	0.81 (0.73, 0.90)		0.004	1.02 (0.96, 1.09)	1.06 (0.96, 1.16)		0.507	0.005

* Adjusted for age, vigorous physical activity category, dietary pattern, job category, and smoking status; ^†^ effects are odds ratios rather than slopes.
